# *Pediococcus pentosaceus* xy46 Can Absorb Zearalenone and Alleviate its Toxicity to the Reproductive Systems of Male Mice

**DOI:** 10.3390/microorganisms7080266

**Published:** 2019-08-16

**Authors:** Shuhua Yang, Ping Gong, Jianwen Pan, Nan Wang, Jingjing Tong, Mingyang Wang, Miao Long, Peng Li, Jianbin He

**Affiliations:** 1 Key Laboratory of Zoonosis of Liaoning Province, College of Animal Science & Veterinary Medicine, Shenyang Agricultural University, Shenyang 110866, China; 2 Institute of Animal Husbandry Quality Standards, Xinjiang Academy of Animal Science, Urumqi 830000, China

**Keywords:** *Pediococcus pentosaceus*, zearalenone, reproductive, toxicity, mice

## Abstract

Zearalenone (ZEA) contamination is a very serious problem around the world as it can induce reproductive disorders in animals and affect the health of humans. Therefore, reducing the damage it causes to humans and animals is a current focus of research. In this study, we assess the removing capacity of *Pediococcus pentosaceus* xy46 towards ZEA and investigate the mechanism responsible for its action, thus confirming if it can alleviate ZEA toxicity to the reproductive systems of male mice. Our results show that the rate at which the strain removes ZEA is as high as 89.2% in 48 h when the concentration of ZEA is 4 μg/mL in the liquid medium. Heat and acid treatment significantly enhanced the ability of the bacteria to remove ZEA. The animal experiments results show that the oral administration of xy46 to mice (0.2 mL daily at a concentration of 10^9^ CFU/mL for 28 days) significantly reduces the degree of testicular pathomorphological changes and apoptosis induced by ZEA when the mice are intragastric administration with 40 mg/kg ZEA daily for 28 days. Moreover, oral administration of xy46 enhances the decrease in the testosterone level and improves the oxidative stress injury induced by ZEA. Furthermore, oral administration of xy46 reverts the expression of these genes and proteins in the testicular tissues of the mice involved in the blood–testis barrier and apoptosis (e.g., Vim, caspase 12, Cldn11, N-cad, Bax, and Bcl-2). However, xy46 cannot significantly revert in some of these evaluated parameters, especially in sperm quantity and quality when the mice were given 70 mg/kg ZEA daily for 28 days. In conclusion, our results suggest that the strain *Pediococcus pentosaceus* xy46 can efficiently remove ZEA from the liquid medium, the mechanism responsible for its action is absorption, and it can alleviate the toxicity of ZEA to the reproductive systems of male mice when the mice are given 40 mg/kg ZEA daily, However, it cannot completely alleviate the reproductive toxicity of higher dosage of zearalenone through its ability to adsorb ZEA.

## 1. Introduction

Mycotoxin contamination is a significant food safety issue around the world. Zearalenone (ZEA) is one of the most prevalent and well-known of these mycotoxins and has estrogen-like effects [1,2,3,4]. ZEA mainly damages the reproductive systems of animals, which causes a decline in livestock and poultry production, resulting in serious economic losses [5,6,7,8]. It is highly resistant to heat treatment (due to the stability of its structure) and must be heated at 225 °C for 30 min to be completely destroyed [9]. It is rapidly absorbed after oral administration, and the biological utilization rate can be over 80% of the intake [10]. Therefore, in order to prevent and treat this problem, it is necessary to identify and administer preventive agents capable of effectively detoxifying the ZEA.

Early studies generally used physical and chemical methods to relieve or attenuate ZEA’s toxicity [11,12,13]. However, such strategies may be aggressive for the environment and significantly change the nutritional value of the feed during the detoxification process. Therefore, bio-detoxification has become an alternative approach that is rising in popularity. Microbial detoxification technology is especially promising as it is harmless to the ecological environment and increases the nutritional value of the feed, thus it is becoming a hot research topic [14,15,16,17,18,19]. As a result, a large number of studies on the detoxification of ZEA by beneficial bacteria have been reported to date [20,21,22,23,24].

Lactic acid bacteria (LAB), which are generally recognized to be safe by the Food and Drug Administration, can inhibit the expansion of Gram-negative pathogens and have a wide range of beneficial effects on host metabolism [25]. LAB also have been used to preserve food products and as feed additives as they can inhibit mycotoxin synthesis and detoxify mycotoxins. Many studies have shown, for example, that *Lactobacillus* spp., such as *L. rhamnosus* [26], *L. plantarum* [27], *L. reuteri* [28], *L. mucosae* [29], and *L. paracasei* [30], can detoxify ZEA. 

*Pediococcus pentosaceus* (*P. pentosaceus*), a facultative anaerobic Gram-positive cocci strain belonging to the LAB family, has many beneficial effects. Studies have shown that *P. pentosaceus* GS4 can protect cadmium (Cd)-induced toxicity in Swiss albino mice [31], *P. pentosaceus* FB145 and FB181 are novel potent biosorbent for preventing cadmium toxicity and reducing its absorption into the human body [32], the oral administration of *P. pentosaceus* LI05 can improve the survival rate and alleviated the histopathological impact of *C. difficile* in mice [33], and *P. pentosaceus* isolated from dairy products can inhibit the growth and zearalenone (ZEA) production of *Fusarium graminearum* [34]. However, *P. pentosaceus* has not been investigated to see if it can remove ZEA and thus alleviate its toxicity to the reproductive systems.

In the present study, the strain *Pediococcus pentosaceus* xy46, which can be isolated from chicken intestines, is assessed to determine its detoxification ability toward ZEA. The mechanism by which it is able to alleviate the toxicity of ZEA to the reproductive systems of male mice is also addressed. It is hoped that this study will thus form the foundation for a new probiotic method of detoxifying ZEA.

## 2. Materials and Methods

### 2.1. Chemicals, Strains, and Media

The ZEA (99.5%) and methanol employed were purchased from Sigma (St. Louis, MO, USA), and the MRS broth was obtained from Hopebio (Qingdao, China). The *Pediococcus pentosaceus* strain xy46 was isolated and preserved by our laboratory, The College of Animal Husbandry and Veterinary Medicine, Shenyang Agricultural University. This strain has been deposited with the China Center for Type Culture Collection and has a preservation number of CCTCC: M2018352.

**Figure A1:**
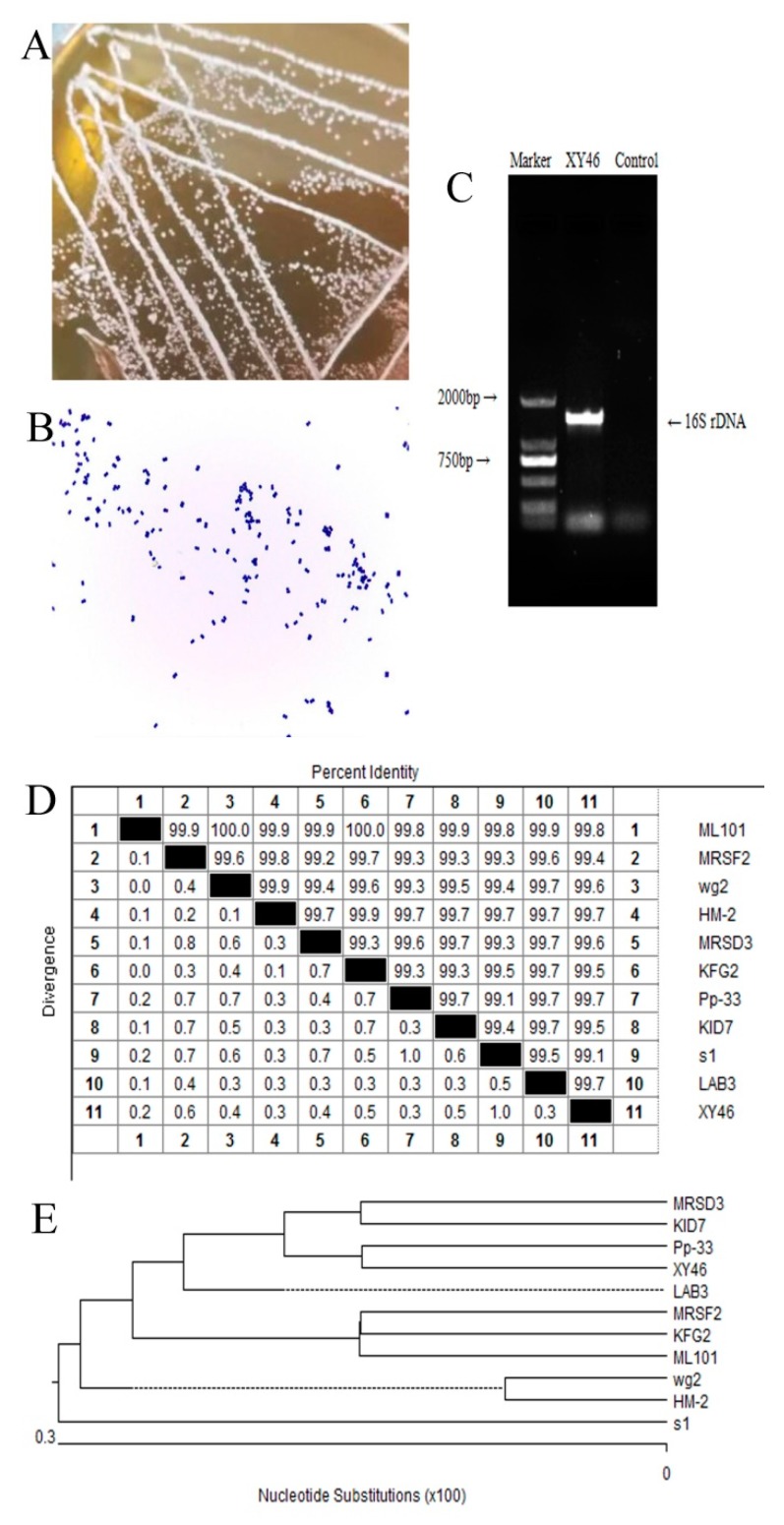
Colony morphology and Gram staining results for the xy46 strain grown on an MRS culture plate, showing: (**A**) the xy46 strain inoculated on an MRS agar plate and anaerobically cultured for 24 h at 37 °C, (**B**) a single colony used for Gram staining, (**C**) electrophoresis results for the 16S rDNA PCR products of xy46, (**D**) 16S rDNA sequence homology analysis of xy46, and (**E**) the phylogenetic tree obtained by comparing the 16S rDNA sequences from the xy46 with the GenBank database.

*Pediococcus pentosaceus* xy46 takes the form of milky-white colonies that are uniform in size, have surfaces that are smooth, and edges that are neat. Spherical or quaternary cells having diameters of 0.8–1.0 μm are revealed by Gram staining and microscopic examination. It is a facultative anaerobic Gram-positive cocci strain. The 16S rDNA sequencing results for the strain (GenBank number: MH424465.1) show that 99% of its DNA sequence is homologous to that of *P. pentosaceus* as listed by the NCBI. The strain was further identified as belonging to *P. pentosaceus* by its physiological (Figure A1A,B) and biochemical characters (Table A1) and by molecular biology identification procedures (Figure A1C–E).

### 2.2. The Ability of Pediococcus pentosaceus xy46 to Remove Zearalenone

The strain was incubated under anaerobic conditions for 24 h at 37 °C. Then, 100 μg (*v/v*) of the strain was inoculated with 4.9 mL of MRS liquid medium containing 4 μg/mL of ZEA and then cultured under the same conditions for another 24 h. The culture was then centrifuged at 3000 *g* for 10 min at 4 °C. The supernatant from 0.5 mL of centrifuged sample was added to an equal volume of chromatography-grade methanol. After 20 min, the liquid was then filtered through a 0.22 organic filter. A 20 μL sample was injected into a high-performance liquid chromatography (HPLC) system with ultra-violet detector and analyzed for ZEA content. The same treatment was repeated with the control group, and the ZEA concentration in the sample was again analyzed using HPLC. Three replicates were done in this test.

The HPLC conditions employed were as follows. Column: Agilent, ZORBAX SB-C18 (4.6 mm × 250 mm, 5 μm); mobile phase: methanol/water (3:1); flow rate: 1 mL/min; sample size: 20 μL; column temperature: 40 °C; ultra-violet detector wavelength = 274 nm. The percentage ZEA removed was calculated using the following formula:

(ZEA peak area in the positive control group − ZEA peak area in the detoxified group)/ZEA peak area in the positive control group × 100%.

### 2.3. Determination of the Removing Mechanism

A method by El-Nezami et al. was used to investigate the mechanism by which the strain removed ZEA [35]. The details of the steps employed were as follows:

Determination of ZEA in supernatant and cell precipitate: The xy46 strain was anaerobically co-cultured with ZEA at 37 °C using a 120 rpm/min shaker for 24 h, the supernatant was extracted by high-speed centrifugation to determine the residual ZEA. At the same time, 80% methanol was added to the cell precipitate to extract ZEA. The distribution and total amount of ZEA in supernatant and cell precipitate of the reaction system were compared.

To further determine whether the strain removes ZEA by adsorption or degradation, the following experiments were conducted. The experimental design and methods are shown in Table 1. The detection methods of ZEA were the same as Section 2.2, and the treated methods of the strain xy46 were as shown in Table 1.

### 2.4. Animals

Male Kunming mice (3 weeks old and 13 ± 2 g in weight) were purchased from Liaoning Changsheng Biotechnology of China. This species/gender was selected because ZEA is an estrogenic mycotoxin that is known to harm their reproductive organs. The use of male mice allows the effect of the ZEA on testis development to be readily observed (testis atrophy and other abnormalities). The mice were maintained under specific pathogen-free (SPF) conditions with restricted access. The humidity was 45–55%, and they were maintained in 12-hour light/dark cycles at a temperature of 23 ± 2 °C. Before the experiment began, the mice were given an acclimatization period of 1 week. The experiments were performed in accordance with the European Communities Council Directive of 24 November 1986 (86/609/EEC) and the principles of SPF laboratory animal care. The experimental procedures employed were approved by the Ethics Committee for Laboratory Animal Care (Animal Ethics Procedures and Guidelines of the People’s Republic of China) for the use of Shenyang Agricultural University, China (PermitionNo. 264 SYXK<Liao>2011-0001, 20 October 2011).

The oral concentration of ZEA employed was determined according to the studies of Long and Wang [36,37]. In addition, the oral concentration of *Pediococcus pentosaceus* used was determined according to the studies of Masuda and Zhao [38,39]. The mice were divided into six groups of 15 mice per group. The characteristics of the treatment groups are shown in Table 2. The mice were intragastric administrated with ZEA after being given xy46 for 2 h at 9:00 every day for 28 days in group xy46 + ZEA40 or xy46 + ZEA70.

### 2.5. Experimental Work

A stock solution of ZEA was prepared in pure alcohol at a concentration of 48 mg/mL and stored at −20 °C. A diluted solution of the xy46 bacterial strain precipitated from physiological saline was also prepared. The xy46 strain was streaked onto MRS agar plates and incubated for 24 h at 37 °C. Single colonies were then inoculated into 5 mL aliquots of MRS medium and incubated for 24 h at 37 °C (150 rpm). Then, 500 μL of a bacterial culture of xy46 cultured for 24 h was inoculated with 50 mL of MRS medium and incubated at 37 °C (150 rpm) for 24 h. After centrifugation at 3000× *g* for 15 min, the precipitated bacterial cells were washed three times with physiological saline and then diluted with physiological saline to 10 mL to form the bacterial solution.

Twenty-four hours after their last treatment, the mice were treated under anesthesia. Blood samples were collected, and the serum was separated. Testicular tissue was collected in cryogenic vials and stored at −80 °C until required for further use.

### 2.6. Physiological Indices and Reproductive Organ Coefficients

The mice were weighed before sacrifice. The weights of the organs were also recorded using an analytical balance after the necropsy. To quantify the effect on the mice, we used an organ coefficient defined according to the equation:

(1) $$\mathrm{Organ}\;\mathrm{coefficient}\;=\;\frac{\mathrm{organ}\;\mathrm{weight}\;\left(g\right)}{\mathrm{mouse}\;\mathrm{body}\;\mathrm{weight}\;\left(g\right)}.$$

### 2.7. Semen Quality Tests

The cauda epididymides of the mice were homogenized in 2 mL of warm (37 °C) saline solution (0.9% NaCl). An aliquot of the diluted spermatozoa suspension was then transferred to a standard hemocytometer counting chamber and allowed to stand for 5 min. A computer-assisted sperm analysis system (Mailang SJ-TMDI810JZ, Nanning Mailang Technology Co., Ltd., Nanning, China) was then used to analyze sperm concentration, percentage of exercise, and rate of deformity.

### 2.8. Testosterone Levels

Plasma testosterone levels were determined by performing enzyme-linked immunosorbent analysis using commercial reagents (total testosterone, MEIMIAN, Wuhan, China). Optical densities (ODs) were measured using a microplate reader (Sunrise-Elisa Reader, Tecan, Mannedorf, Switzerland) and used to determine standard curves and sample concentrations. In this work, the results are expressed in ng/mL serum.

### 2.9. Antioxidant Stress Index

We determined the levels of malondialdehyde (MDA), catalase (CAT), glutathione peroxidase (GSH-Px), total superoxide dismutase (T-SOD) in the tissue using thiobarbituric acid (TBA), visible light, colorimetric, and hydroxylamine methods, respectively, using commercially available reagents (JianCheng, Nanjing, China). The ODs were measured using a microplate reader (Sunrise-Elisa Reader, Tecan, Mannedorf, Switzerland) and used to determine standard curves and sample concentrations.

### 2.10. Histopathological Variation in the Testes

Before conducting routine processing and paraffin embedding, the testis tissues were set in 10% formalin. We then used hematoxylin and eosin (HE) to stain the testis sections and imaged them using a photomicroscope (Nikon, Eclipse E100, Tokyo, Japan).

### 2.11. TUNEL Staining

Paraffin sections were dewaxed and hydrated. After washing with PBS, 3 testicular tissue paraffin sections from each group were subjected to TUNEL staining. The sections were then inspected under a fluorescence microscope (Olympus BX61, Chiba-ken, Japan) and images collected.

### 2.12. Gene Expression

Total RNA was extracted from the testicular tissues using RNAiso Plus reagent (9108, Applied TaKaRa, Dalian, China). The purity of the total RNA was measured using a microvolume spectrometer (uLITE, Biochrom Ltd. Cambridge, UK) at an absorbance ratio of 260/280 nm, and then the total RNA extracted was reverse transcribed using a TaKaRa PrimeScript^TM^ RT kit (RR047A, Applied TaKaRa, Dalian, China) and the cDNA recorded. A value in the range 1.8–2.0 indicates that the RNA sample is pure.

Real-time polymerase chain reaction (RT-PCR) was performed using an ABI 7500 RT-PCR system and a SYBR^®^ Premix Ex Taq^TM^ II kit (RR820A, Applied TaKaRa, Dalian, China). In the quantitative RT-PCR (qRT-PCR) experiments, the total volume of the PCR reaction mixture was 20 μL, which consisted of 2 μL of cDNA product, 0.8 μL of reverse primer, 0.8 μL of forward primer, 10 μL of Taq Master Mix solution, 6 μL RNase-free water, and 0.4 μL of Rox. PCR was carried out as follows: An initial denaturation step (95 °C for 30 s), then 40 cycles, 5 s at 95 °C, 34 s at 60 °C, and 15 relative changes at 95 °C. The mRNA values were calculated using the 2^−ΔΔCt^ method. The primers used, which were designed and synthesized by Sangon (Shanghai, China), are shown in Table 3.

### 2.13. Western Blot Analyses

The total protein was obtained from testicular tissue using a ProteinExt^®^ Mammal Total Protein Extraction Kit (DE101, TransGen Biotech, Beijing, China). An Easy II Protein Quantitative Kit was used to determine the protein concentration (DQ111, TransGen Biotech, Beijing, China). The proteins were separated via SDS separation using polyacrylamide gel electrophoresis and transferred to PVDF membranes (Solarbio, Beijing, China). The membranes were cultured overnight at 37 °C and then with antibodies below 4 °C.

The membranes were washed with TBST (1 L of TBST contains 50 mL of TrisHCL (1 M, pH 7.5); 8 g of NaCl; 0.2 of KCL; 0.5 mL of Twain) and incubated with a secondary antibody blocking solution for 2 h at room temperature [40]. Proteins were detected using a DNR Bio Imaging system using the NcmECL Ultra method according to the manufacturer’s instructions (Ncmbio, Suzhou, China). The expression of target proteins was quantified using a gel quantification system.

### 2.14. Statistical Analyses

All the animal experiments were repeated at least three times independently. At least three mice were taken from each experimental group for testing. All experimental data were initially calculated by Excel and then analyzed by one-way ANOVA using data analysis software SPSS17.0. The experimental values were expressed as mean ± SD, *p* < 0.05 was statistically significant, which was indicated that the differences between the test groups are significant. All the results and charts in this experiment were calculated and drawn by Graphpad Prism 5 and Microsoft Office.

## 3. Results

### 3.1. Ability of xy46 to Remove ZEA

**Figure 1:**
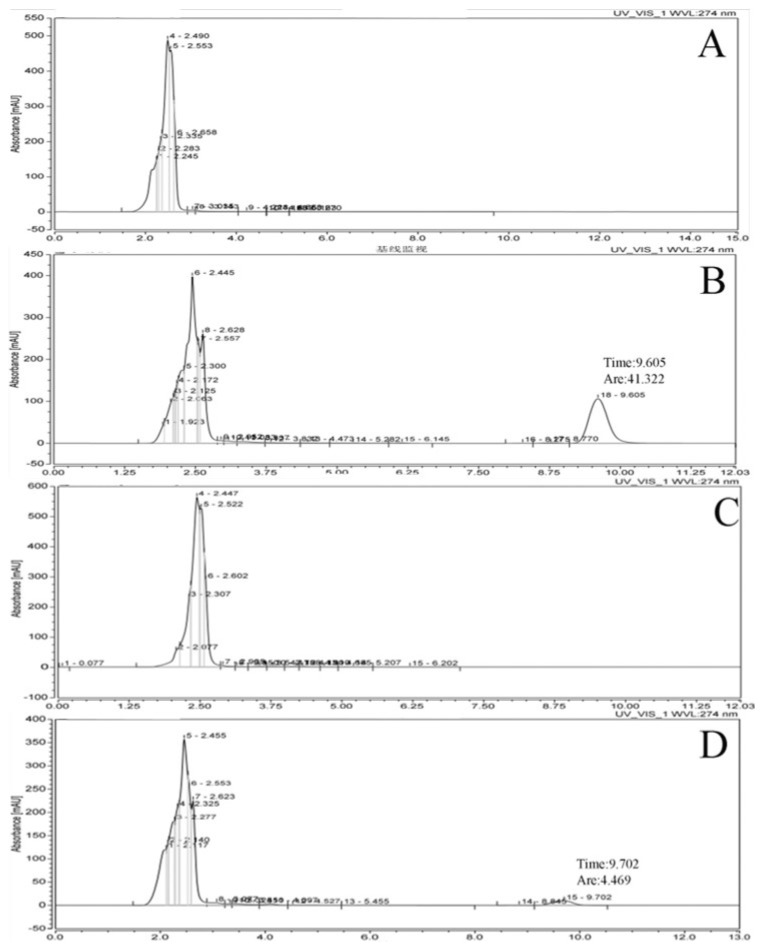
Determining the ability of *Pediococcus pentosaceus* xy46 to remove ZEA via high performance liquid-phase detection. The chromatograms correspond to the HPLC results obtained for: (**A**) the solvent control group, (**B**) the ZEA group, (**C**) the bacterial liquid control group, and (**D**) the xy46 + ZEA group. The abscissae values correspond to the retention times of the detected substance and the ordinates to the peak absorbance intensity in each time period.

The HPLC results are shown in Figure 1. As can be seen, the solvent has a retention time of 2–4 min; that of ZEA is 9–10 min. The peak in the xy46 + ZEA group is about 10 times smaller than that in the ZEA group, which indicates that the *P. pentosaceus* xy46 strain has a good ability to absorb ZEA. A calculation using the expression given earlier implies that the removing rate is around 89.2%.

### 3.2. Removing Mechanism

**Figure 2:**
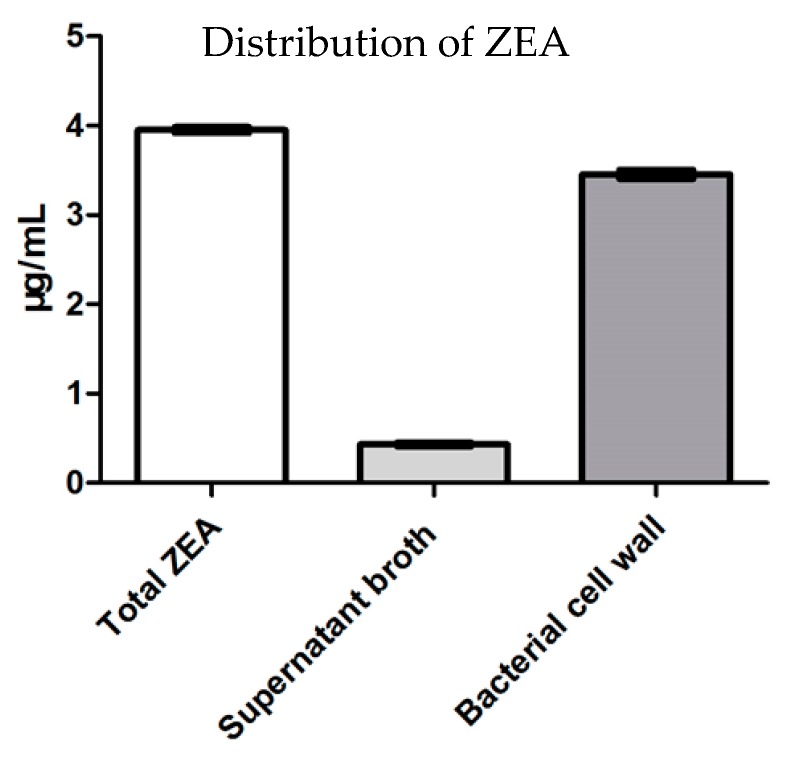
The contents of ZEA in supernatant and cell precipitate after the xy46 strain was anaerobically co-cultured with ZEA at 37 °C using a 120 rpm/min shaker for 24 h. The experiments are averages of three replicates.

Figure 2 shows the contents of ZEA in supernatant and cell precipitate. The results show after ZEA co-treated with the xy46 strain for 24 h, the content of ZEA in the supernatant is 0.432 μg/mL, and the content of ZEA in precipitate is 3.456 μg/mL. The total content of ZEA in the extract of cell precipitate of xy46 and in the medium supernatant is about 4 μg/mL. That is, ZEA added to the culture media was completely distributed between the supernatant and the cell precipitate. The total amount of ZEA in the experiment did not decrease. If ZEA was degraded by the strain, the total amount of ZEA would decrease according to the law of conservation of mass. Thus, xy46 removes ZEA in the solution by adsorption.

Table 4 shows the removing rates obtained using the different methods. The table shows that when using the live xy46 strain, the rate of ZEA removal was higher in the short-term co-cultivation group compared to the long-term co-cultivation group (*p* < 0.05). In addition, the inactivated groups (acid or heat-treated) had better results than those of the live bacteria groups (the short-term co-cultivation group and the long-term co-cultivation group) (*p* < 0.05). Clearly, heat and acid treatment significantly enhanced the ability of the bacteria to remove ZEA.

These results must be combined with the information shown in Figure 2. The total contents of ZEA in supernatant and cell precipitate after the xy46 strain was anaerobically co-cultured with ZEA did not change during this process. Thus, the mechanism by which *P. pentosaceus* xy46 removes ZEA is adsorption, not degradation.

### 3.3. Physiological Indices and Reproductive Organ Coefficients

**Figure 3:**
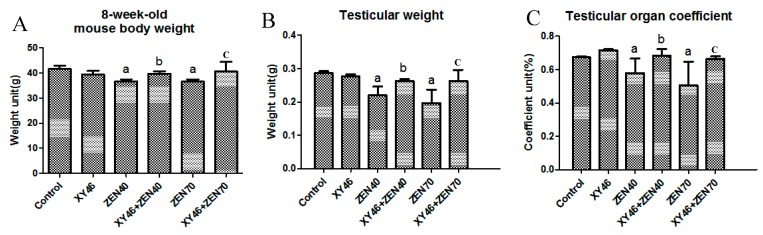
Mouse physiology and reproductive organ indices, showing: (**A**) mouse body weights after 8 weeks, (**B**) mouse testicular weights after 8 weeks, and (**C**) the corresponding mouse reproductive organ coefficients. Key: “a”—result is significantly different compared to the control group (*p* < 0.05), “b”—xy46 + ZEA40 is significantly different from the ZEA40 result (*p* < 0.05), and “c”—xy46 + ZEA70 result is significantly different from the ZEA70 result (*p* < 0.05). Seven mice were randomly selected from each group for testing.

Figure 3 shows the body/testicular weights and genital indices obtained when the mice were 8 weeks old. It is clear that the mice in the ZEA40 and ZEA70 groups were significantly lighter than those in the control group (*p* < 0.05). In addition, the mice in the xy46 + ZEA40, ZEA40, and xy46 + ZEA70 groups were significantly heavier than those in the ZEA70 group (*p* < 0.05). The results therefore show that ZEA reduces the body/testicular weights and organ indices in mice, and that co-treating them with *P. pentosaceus* xy46 significantly reduces the harmful effect of the ZEA on reproductive organ.

**Figure 4:**
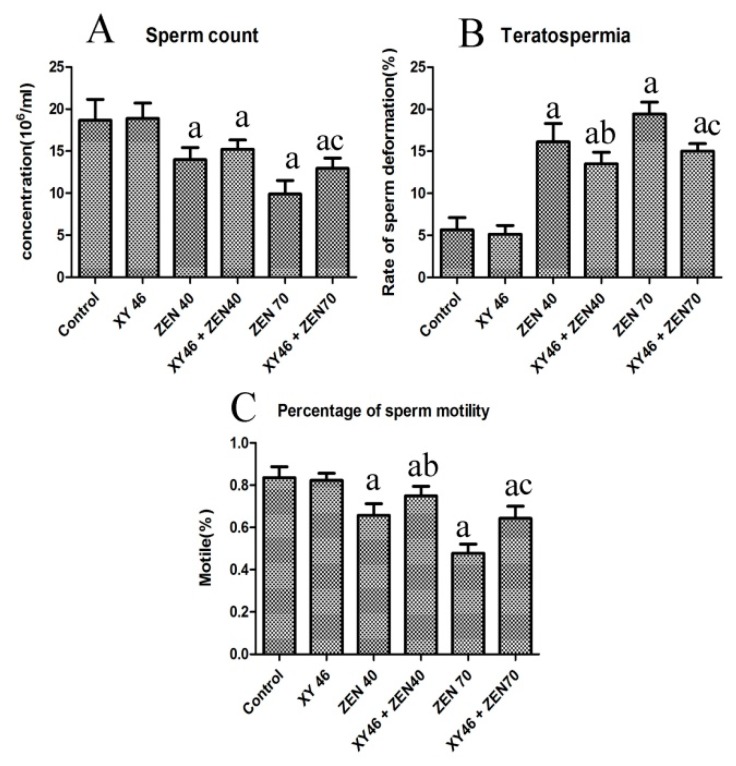
Plots highlighting the quality of the semen derived from the mice, showing: (**A**) mouse sperm concentration, (**B**) mouse sperm deformity rate, and (**C**) sperm motility rate. Key: ‘a’—significantly different compared to the control group (*p* < 0.05), ‘b’—xy46 + ZEA40 is significantly different from the ZEA40 group (*p* < 0.05), and ‘c’—significant difference between xy46 + ZEA70 and ZEA70 groups (*p* < 0.05). Seven mice were randomly selected from each group for testing.

**Figure 5:**
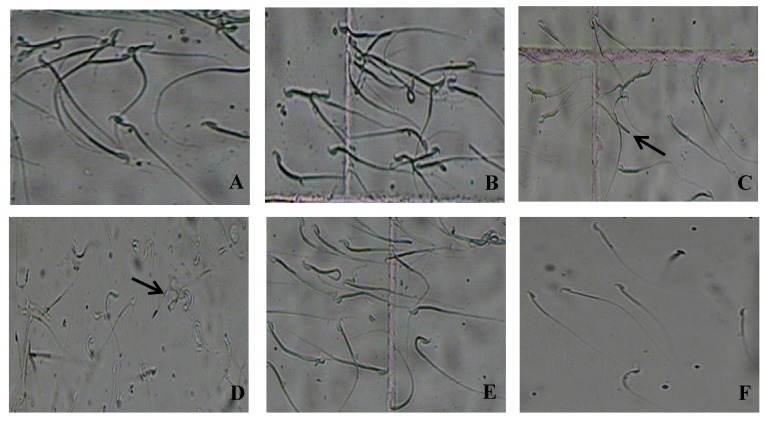
Photographs of mouse sperm showing the types of deformity observed: (**A**) control group, (**B**) detoxifying bacteria group (xy46), (**C**) low-toxicity group (ZEA40), (**D**) high-toxicity group (ZEA70), (**E**) low-toxicity detoxified group (xy46 + ZEA40), and (**F**) high-toxicity detoxified group (xy46 + ZEA70). In (**C**), the arrow highlights sperm that has a head but no hook, a double head, and is amorphous. In (**D**), the arrow shows the tail of a sperm that is becoming folded.

Figure 4 shows further information about the sperm obtained from the subjects in the different groups. Clearly, the concentrations and exercise rates of the mice sperm in the ZEA40 and ZEA70 groups are significantly smaller than those in the control group, but the deformity rates are higher (*p* < 0.05). The percentage of sperm motility and teratospermia in the xy46 + ZEA40 group and xy46 + ZEA70 are effectively reversed if we compare with that in the ZEA40 and ZEA70 groups (*p* < 0.05). The mouse sperm was clearly affected by the ZEA and many suffered head and tail deformities (Figure 5).

The results show that the mice semen has its quality significantly reduced by exposure to ZEA. More importantly, the administration of *P. pentosaceus* xy46 is able to significantly improve the quality of the semen in mice resulting from such exposure although it is not restored to the same level as the control group.

### 3.4. Pathological Tissue Sections

**Figure 6:**
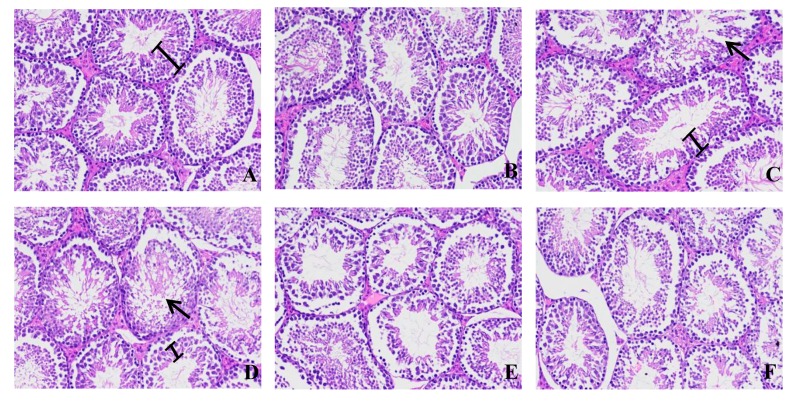
Sections of HE-stained mice testes in paraffin, showing: (**A**) control group, (**B**) detoxifying bacteria group (xy46), (**C**) low-toxicity group (ZEA40), (**D**) high-toxicity group (ZEA70), (**E**) low-toxicity detoxified group (xy46 + ZEA40), and (**F**) high-toxicity detoxified group (xy46 + ZEA70). The arrows in (**C**,**D**) indicate loosely released spermatogenic cells. The thickness of the spermatogenic epithelium is also compared in (**A**,**C**,**D**) which suggests that higher ZEA concentrations lead to thinner spermatogenic epithelia. In each picture, the magnification is ×200.

Figure 6 presents images of the HE-stained sections of the mouse testes. We can see that a large number of spermatogenic cells in the ZEA40 and ZEA70 groups were loose, or even detached, compared to the control group. This would result in their separation from the supporting cells, leading to a significant thinning of the sperm. The epithelial thickness and number of cells undergoing spermatogenesis is thus greatly reduced.

The images obtained from the xy46 + ZEA40 group show that the seminiferous epithelium in this group is significantly increased in thickness (compared to the ZEA40, xy46 + ZEA70, and ZEA70 groups), and the connection between the seminiferous tubules is tighter. These results thus show that *P. pentosaceus* xy46 is effectively able to alleviate the damage that ZEA causes to the mouse testes.

### 3.5. Oxidative Parameters and Serum Testosterone Levels

**Figure 7:**
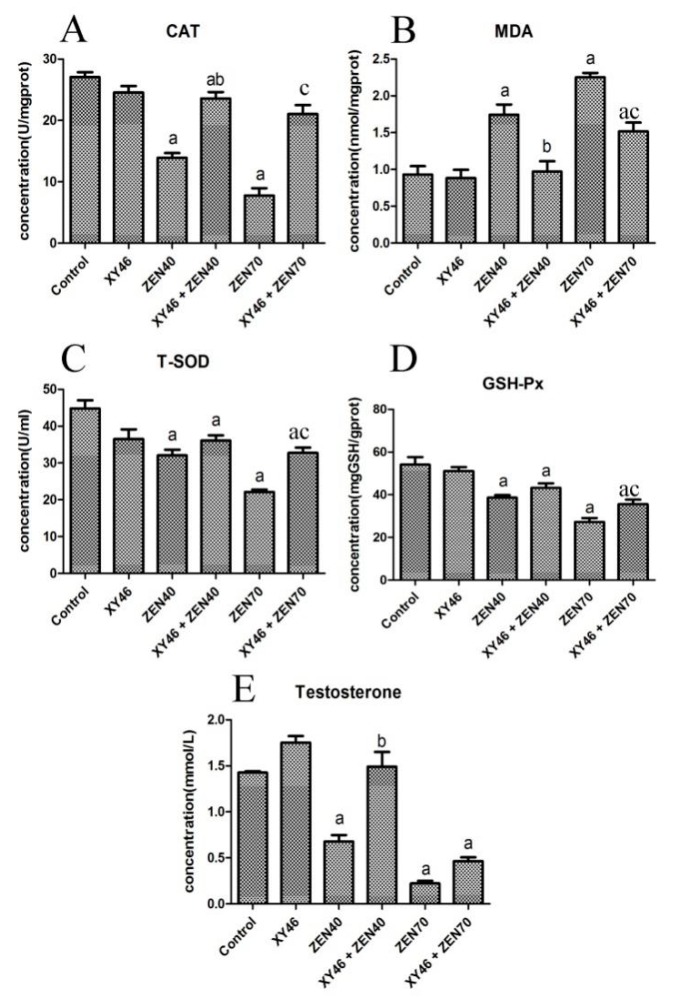
The detected concentrations of substances indicating oxidative damage to the mice testes and the concentration of testosterone in the mice serum, showing: (**A**) representative CAT levels in the testicular tissue, (**B**) MDA levels, (**C**) T-SOD levels, (**D**) GSH-Px levels, and (**E**) testosterone levels in the serum. Key: ‘a’—significantly different result compared to the control group (*p* < 0.05), ‘b’—xy46 + ZEA40 result that is significantly different from the ZEA40 result (*p* < 0.05), and ‘c’—xy46 + ZEA70 result that is significantly different from the other groups (*p* < 0.05). Seven mice were randomly selected from each group for testing.

Figure 7A–D shows that the MDA content in the ZEA40 and ZEA70 groups is increased (compared to the control group, *p* < 0.05), while the CAT, T-SOD, and GSH-Px activity in the mouse testes were all decreased. However, these changes were all reversed (to differing degrees) when the mice were co-treated with the xy46 strain (*p* < 0.05), but for high concentration of ZEA (70 mg/kg ZEA) the levels of evaluated parameters are not reestablished by given *P. pentosaceus* xy46. The results thus indicate that co-treating the mice with *P. pentosaceus* xy46 significantly reduces the oxidative damage caused by the toxic ZEA at lower concentration (40 mg/kg ZEA).

Figure 7E shows the test results obtained for testosterone in the mice serum. We can see that the testosterone content in the ZEA40 and ZEA70 groups is significantly lower than that in the control group (*p* < 0.05). However, the testosterone content in the xy46 + ZEA40 group is significantly increased compared to those in the ZEA40 group (*p* < 0.05), but for high concentration of ZEA (70 mg/kg ZEA) the testosterone levels are not reestablished by given *P. pentosaceus* xy46. The results therefore show that the ZEA reduces the production of testosterone in mice and that co-treating them with *P. pentosaceus* xy46 significantly reduces the harmful effect of the ZEA when giving the mice 40 mg/kg ZEA. However, *P. pentosaceus* xy46 cannot reduce the harmful effect of the ZEA when giving the mice 70 mg/kg ZEA.

### 3.6. Genes, Proteins, and TUNEL Results

**Figure 8:**
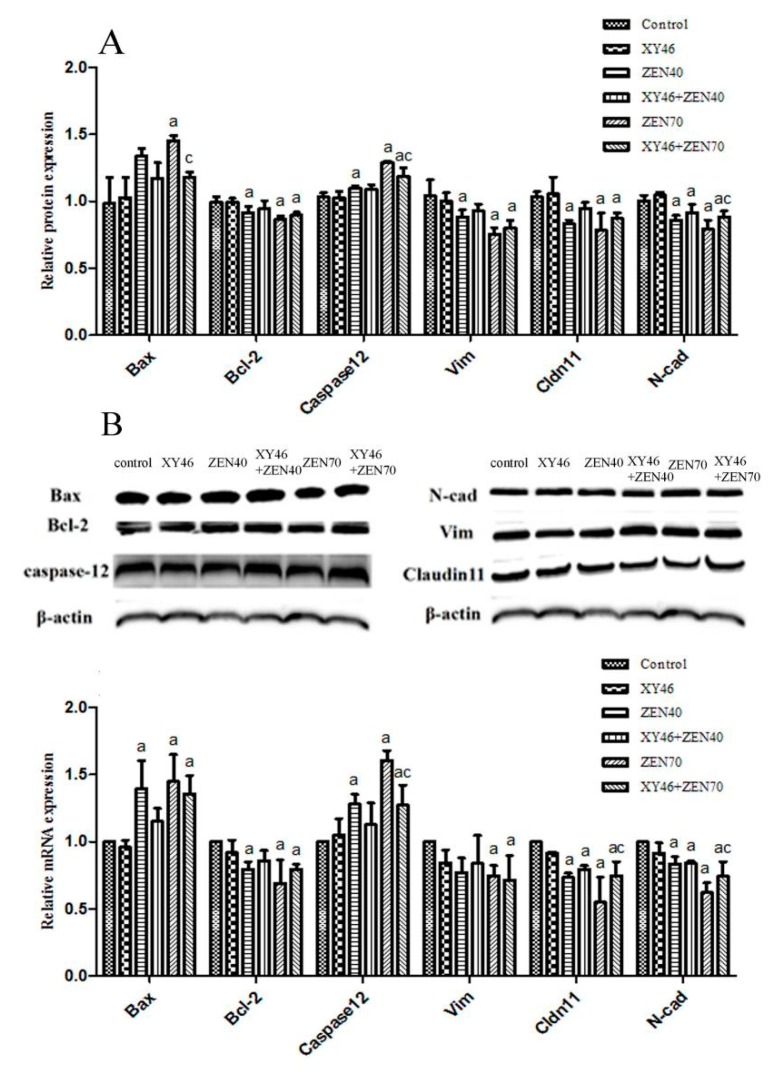
The results for mRNA and protein expression in mouse testes, showing: (**A**) protein expression levels, (**B**) mRNA expression levels. Key: ‘a’—significantly different compared to the control group (*p* < 0.05), ‘b’—significantly different xy46 + ZEA40 result to the ZEA40 result (*p* < 0.05), and ‘c’—significantly different results between xy46 + ZEA70 and ZEA70 groups (*p* < 0.05).

Figure 8 shows the results obtained for protein and mRNA expression. Compared with the control group, the ZEA significantly increases the protein and mRNA expression of the proapoptotic genes Bax and Caspase 12 (*p* < 0.05) and significantly down-regulates the expression of the antiapoptotic gene Bcl-2. However, compared to the ZEA70 group, the xy46 + ZEA70 group experienced lower levels of Bax protein expression (*p* < 0.05) and Caspase 12 protein and mRNA expression (*p* < 0.05). Thus, the results show that the *P. pentosaceus* significantly attenuates the level of ZEA-induced apoptosis in the testicular tissue cells.

The ZEA also significantly decreased the protein and mRNA expression levels of Vim, Cldn 11, and N-cad in the ZEA40 and ZEA70 groups compared to the control group (*p* < 0.05). Compared to the ZEA70 group, however, the xy46 + ZEA70 group has increased protein and mRNA expression levels of Cldn 11 and N-cad (*p* < 0.05). Thus, the xy46 strain has an important role to play in protecting the testicular Sertoli cells against ZEA-induced tight-junction injuries.

**Figure 9:**
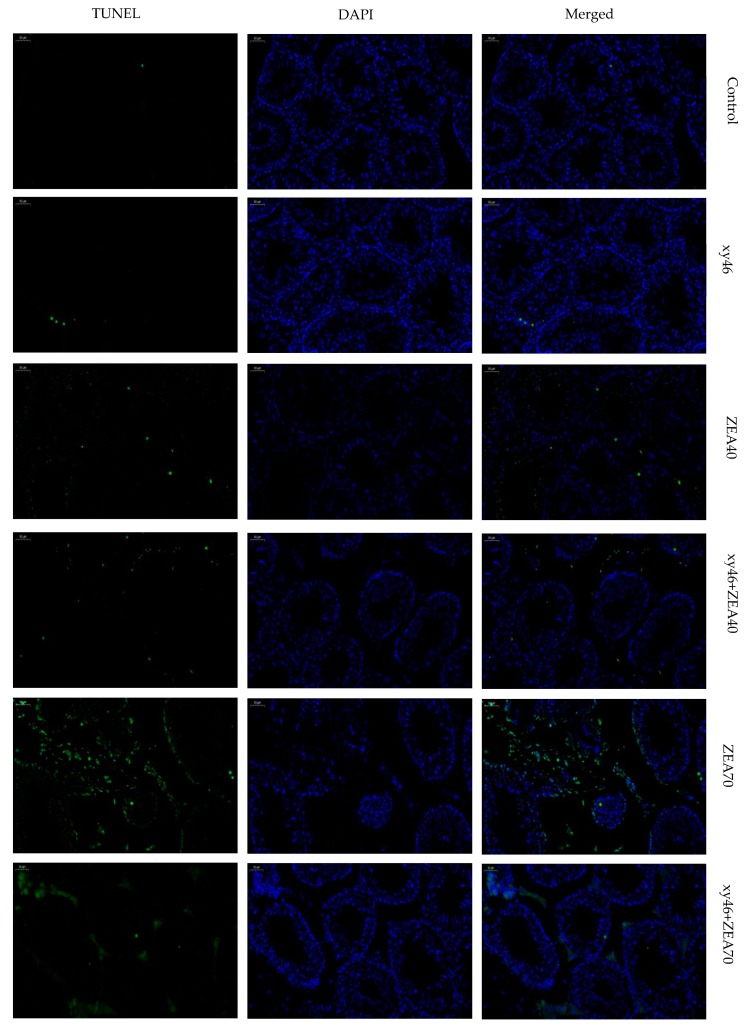
Results of TUNEL staining of mouse testis tissue. Green fluorescence indicates TUNEL-positive cells. DAPI was used for nuclear staining in the microscopic fields. Magnification is 200×.

At the same time, the TUNEL results (Figure 9) show that the number of apoptotic cells in the ZEA40 and ZEA70 groups was significantly increased compared to the control group. However, the number of apoptotic cells is significantly lower in the xy46 + ZEA40 and xy46 + ZEA70 groups. These results also show that co-treating the mice with *P. pentosaceus* xy46 significantly reduces the pathological damage and apoptosis of mouse testis cells induced by ZEA.

## 4. Discussion

Previous studies have shown that many LAB species are able to detoxify ZEA. However, it has not been reported whether or not *Pediococcus pentosaceus* has this ability. Our study shows that *P. pentosaceus* xy46 is able to efficiently remove ZEA, which means that there is a new strain available for the microbial detoxification of ZEA.

At present, there are two main ways of achieving the microbial detoxification of ZEA [24]: Degrade the mycotoxin by transforming it [20,21], or reduce the bioavailability of the mycotoxin by adsorbing it [22,23]. In order to clarify the mechanism by which xy46 removes ZEA, the total amount of ZEA in supernatant and cell precipitate was detected, the removal rate of ZEA was examined when the strain xy46 interacted with ZEA over a long time or a short time, and the ability of the strain xy46 treated by acid/heat to remove ZEA was also detected. Our results shows that the total amount of ZEA does not decrease. This results might be due to the fact that: Firstly, ZEA is relatively stable under this experimental condition, and does not degrade, but distributes in supernatant and cell precipitate; second, ZEA is adsorbed on the cell surface, which can be extracted by adding 80% methanol extraction. The time required for the conversion (degradation) process is generally longer than that required for the adsorption (binding) process. This is because, in the former case, it takes time for cells to produce certain specific enzymes, and there is also the time required for the enzymes to react with the ZEA as well. On the other hand, there is only adsorption involved in the binding method, which does not require a large amount of reaction time [35]. T Overall, according to our results, it can be concluded that the removing mechanism by which *P. pentosaceus* xy46 neutralizes ZEA is adsorption (binding).

Although many studies have confirmed the removing ZEA ability of probiotics in vitro, very few studies have demonstrated in vivo that probiotics can reduce the toxicity of toxins to animals by removing toxins. The present study has examined the ability of xy46 to counteract the reproductive damage induced by ZEA in mice from four points of view: Whole structure, tissue structure, cell structure, and molecular level.

At the whole-structure level (considering the changes determined in reproductive organ coefficient, weight change per week, and feed intake of the mice), we can conclude that ZEA produces toxicity in mice after 28 days of continuous intragastric administration. At the histological structure level (according to our pathological and TUNEL staining results), we found that ZEA mainly damages the Sertoli cells, which is consistent with previous findings [41,42,43].

On a more cellular level, we have confirmed that the ZEA affects the ability of the testicular cells to scavenge oxidative free radicals and hydrogen peroxide. It has a very toxic effect on the cells, and even affects the structure and function of the cell membranes [44,45]. However, co-treating the subject with the xy46 strain significantly reverted these indicators when the mice were given 40 mg/kg ZEA, however, the strain xy46 could not significantly revert these indicators when the mice were given 40 mg/kg ZEA. Thus, the *P. pentosaceus* xy46 effectively reduced the toxicity of ZEA towards the reproductive cells of the mice when the mice were given 70 mg/kg ZEA.

At the molecular level, our results show that the protein and mRNA expression levels of Bax and Caspase 12 are significantly increased after mice are exposed to ZEA, and the Bcl-2 level is significantly decreased in the testis tissues, consistent with the results of previous studies [46,47,48]. Our study also shows that the oral administration of ZEA causes the down-regulation of the expression of Vim, Cldn 11, and N-cad in the mouse testis tissues, again consistent with the results of a previous study [36]. However, co-treatment with the strain xy46 significantly reverts these indicators when the mice are given 40 mg/kg ZEA. On the other hand, the strain xy46 could not significantly revert these indicators when the mice were given 70 mg/kg ZEA. This demonstrates that *P. pentosaceus* xy46 effectively reduced the toxicity of ZEA to the reproductive organs in mice at the molecular level as well when the mice were given 40 mg/kg ZEA. 

## 5. Conclusions

This study is the first to report that *Pediococcus pentosaceus* can effectively absorb zearalenone in liquids and to show that the treatment of male mice with the strain *Pediococcus pentosaceus* xy46 can effectively alleviate the toxic effects induced in their reproductive organs by ZEA when the mice are given 40 mg/kg ZEA daily. However, *P. pentosaceus* xy46 cannot completely alleviate the reproductive toxicity of higher dosage of zearalenone through its ability to adsorb ZEA. This study therefore lays a foundation for the future use of *Pediococcus pentosaceus* xy46 as a food additive to alleviate zearalenone toxicity to animals.

## Figures and Tables

**Table 1:** The experimental design of the xy46 strain treated in different ways then co-cultivated with zearalenone (ZEA).

Group	Methods
Short-term co-cultivation group	The xy46 strain was cultivated in MRS liquid medium for 24 h and then co-cultivated with ZEA for 15–30 min
Long-term co-cultivation group	Both the xy46 strain and ZEA were co-cultivated at the same time in MRS liquid medium for 24 h
Heat treatment group	The xy46 strain was cultivated in MRS liquid medium for 24 h, then autoclaved at 121 °C for 30 min, and then co-cultivated with ZEA for 15–30 min
Acid treatment group	The xy46 strain was cultivated in MRS liquid medium for 24 h, then treated with hydrochloric acid (the final pH was about 1) and then co-cultivated with ZEA for 15–30 min

**Table 2:** Animal test design.

Group	Substance Administrated	Intragastric Administration/Single	Time
Control	0.9% NaCl	0.2 mL	Daily for 28 days
xy46	10^9^ CFU/mL	0.2 mL	Daily for 28 days
ZEA40	40 mg/kg ZEA	0.2 mL	Daily for 28 days
xy46 + ZEA40	40 mg/kg ZEA + 10^9^ CFU/mL xy46	0.2 mL	Daily for 28 days
ZEA70	70 mg/kg ZEA	0.2 mL	Daily for 28 days
xy46 + ZEA70	70 mg/kg ZEA + 10^9^ CFU/mL xy46	0.2 mL	Daily for 28 days

**Table 3:** Primer sequences.

Gene	Serial Number	Primer Sequence (5′—3′)	Product Length
*Bax*	NM_007527.3	Forward: TCCACCAAGAAGCTGAGCGAG Reverse: GTCCAGCCCATGATGGTTCT	257 bp
*Bcl-2*	NM_009741.5	Forward: GACAACGGAGGATGGGATG Reverse: TCCACGATA AACTGGGTGACT	I50 bp
*Caspase12*	NM_009808.4	Forward: CTCAATAGTGGGCATCTGGGT Reverse: GAAGGTAGGCAAGACTGGTTC	151 bp
*β-actin*	BC_138614.1	Forward: CTGTCCCTGTATGCCTCTG Reverse: TTGATGTCACGCACGATT	221 bp
*Vim*	NM_011701.4	Forward: GATCAGCTCACCAACGACAA Reverse: GCTTTCGGCTTCCTCTCTCT	120 bp
*Cldn 11*	NM_008770.3	Forward: GGGTGCTCCTTATTCTGCTG Reverse: AGCGAGTAGCCAAAGCTCAC	103 bp
*N-cad*	AB_008811.1	Forward: AGGACCCTTTCCTCAAGAGC Reverse: ATAATGAAGATGCCCGTTGG	117 bp

**Table 4:** ZEA removing rates obtained using differently treated methods.

Groups	ZEA Removing Rate (%)
Short-term co-cultivation group	60.4 ± 1.064 ^a^
Long-term co-cultivation group	55.9 ± 0.56 ^b^
Heat treatment group	94.4 ± 2.13 ^c^
Acid treatment group	80.0 ± 2.54 ^d^

The difference letters in the upper corner mean significant difference between the two groups (*p* < 0.05).

## Appendix

**Table A1:** Biochemical identification results for xy46.

Identification Project	Result
Aerobic culture	+
Anaerobic culture	+
pH 1.5	+
pH 2.5	+
pH 3.5	+
pH 9.6	−
Glucose produces acid and gas?	Produces acid but not gas
Mannitol	−
Lactose	+
Sucrose	+
Maltose	+
Xylose	−
Fructose	−
Mannose	−
Galactose	−
